# Chinese Medicine* FTZ* Recipe Protects against High-Glucose-Induced Beta Cell Injury through Alleviating Oxidative Stress

**DOI:** 10.1155/2019/6378786

**Published:** 2019-03-03

**Authors:** Weijian Bei, Yujiao Wang, Jianmei Chen, Jingjing Zhang, Lexun Wang, Zhanhui Gu, Yinming Hu, Yijian Huang, Wei Xu, Zili Lei, Jinyan Cai, Jiao Guo

**Affiliations:** ^1^Guangdong Metabolic Disease Research Center of Integrated Chinese and Western Medicine, Guangdong TCM Key Laboratory against Metabolic Diseases, Key Unit of Modulating Liver to Treat Hyperlipemia SATCM (State Administration of Traditional Chinese Medicine), SATCM Level 3 Lab of Lipid Metabolism, Institute of Chinese Medicinal Sciences, Guangdong Pharmaceutical University, Guangzhou Higher Education Mega Center, Guangzhou, 510006, China; ^2^Guangzhou University of Chinese Medicine, Guangzhou, China

## Abstract

**Objective:**

To investigate the effect of FTZ on high-glucose-induced oxidative stress and underlying mechanisms.

**Methods:**

We used a *β* cell dysfunction and diabetes model that was induced in rats fed a high-fat high-sugar diet (HFHSD) for 6 weeks and injected once with 35 mg/kg streptozocin (STZ). Then, 3 and 6 g/kg of FTZ were administered by gavage for 8 weeks. In addition, an ex vivo model of oxidative stress was induced by stimulating INS-1 cells with 25 mmol/L glucose for 48 h.

**Result:**

The levels of fasting blood glucose (FBG) in diabetic model rats were obviously higher than those in the normal group; furthermore with reduced levels of *β* cells, catalase (CAT), superoxide dismutase (SOD), and Bcl-2 increased lipid peroxide malondialdehyde (MDA) and caspase-3 in the pancreatic tissue of the diabetic model rats. Afterward, the cells were incubated with FTZ-containing serum and edaravone. The 25 mmol/L glucose-induced SOD reduction increased MDA and intracellular ROS. The protein expression level of Mn-SOD and CAT in the model group decreased significantly compared with that in the control group.

**Conclusion:**

FTZ treatment significantly improved the alteration in the level of SOD, CAT, Bcl-2, caspase-3, and MDA coupled with *β* cell dysfunction in diabetic rats. Oxidative stress in INS-1 cells was closely associated with a higher rate of apoptosis, increased production of ROS and MDA, enhanced Bax expression, and caspase-3, -9 activities and markedly decreased protein expression of Mn-SOD and CAT. FTZ-containing serum incubation notably reversed the high-glucose-evoked increase in cell apoptosis, production of ROS and MDA, and Bax protein levels. Furthermore, FTZ stimulation upregulated the expression levels of several genes, including Mn-SOD, CAT, and Bcl-2/Bcl-xl. In addition, FTZ decreased the intracellular activity of caspase-3, -9 in INS-1 cells. FTZ protected *β*-cells from oxidative stress induced by high glucose in vivo and in vitro. The beneficial effect of FTZ was closely associated with a decrease in the activity of caspase-3, -9 and intracellular production of ROS, MDA, and Bax coupled with an increase in the expression of Mn-SOD, CAT, and Bcl-2/Bcl-xl.

## 1. Introduction

Type 2 diabetes, a chronic metabolic noncommunicable disease characterized by high blood glucose, with pathologic insulin resistance and subsequent *β*-cell dysfunction, has become a serious hazard to human health [1, 2]. Oxidative stress resulting from exposure to high level glucose is considered to be one of the key factors of *β*-cell dysfunction in type 2 diabetes and the basis of diabetic complications [3–10].

Oxidative stress refers to the imbalance between the production and removal of reactive oxygen species (ROS), which leads to the gradual accumulation of ROS in the body and damage of biological macromolecules such as proteins, nucleic acids, and lipids in the body [5]. Under constantly high blood glucose levels, superoxide products will increase greatly, and oxidative stress will be generated when the production rate of the superoxide products exceeds the removal rate [6–8]. Chronic hyperglycemia is mainly accountable for the oxidative stress, which is the major cause of increased oxidative stress damage and can cause islet *β* cell function injury and peripheral insulin resistance, increasing the risk of diabetes [8–14]. Oxidative stress and ROS cause islet *β* cell damage through the NF-*κ*B pathway [15, 16], which prompts follow-up apoptosis signaling cascade amplification, leading to cell apoptosis [17, 18].

Oxidative stress can aggravate insulin resistance and cause islet *β* cell damage by inducing cascade reactions of various serine kinases, interfering with the phosphorylation of insulin receptors (InsRs) and insulin receptor substrate (IRS) [19], and by activating the NF-*κ*B pathway [15], JNK/SAPK pathway [16], p38 MAPK pathway [16], and hexosamine pathway [19], which prompts follow-up apoptosis signaling cascade amplification, leading to cell apoptosis.

Antioxidative treatment will play a larger role in the treatment of diabetes and has a promising future, which may increase intracellular antioxidative agents such as catalase (CAT) and superoxide dismutase (SOD) and eliminate lipid peroxide malondialdehyde (MDA)[3, 4, 6, 13, 20, 21]. It has been reported that several traditional Chinese medicines (TCMs) have been effectively used for treating diabetes [1, 2, 22].

Currently, oral administrative drugs commonly used in the treatment of type 2 diabetes mainly include insulin secretion agents (sulfonylurea and glycine secretion), insulin sensitizers (biguanide and gliadin), and alpha-glycosidases[1, 20]. However, most of the glucose-lowering drugs for the treatment of diabetes show obvious side effects such as stoma, anorexia, nausea, vomiting, and diarrhea. Moreover, liver or kidney damage occurs in some patients after long-term usage [1, 20, 23]. TCM dialectical treatment characterized by compatibility and flexible, multicomponent, multitarget, and multilink adjustment has great advantages and lower side effects and is a promising choice for diabetes treatment [21, 22, 24].

Fufang Zhenshu Tiao-Zhi (FTZ) recipe capsules are composed of eight traditional Chinese medicinal herbs, such as Buddhist, Huanglian, Nvzhenzi, and Sanqi [24]. Our previous studies have shown that FTZ improves hyperglycemia and hyperlipidemia, alleviates inflammation, regulates blood coagulation, and protects the vascular endothelium [22, 24–26]. In addition, FTZ regulates multiple links of lipid metabolism, such as absorption, synthesis, transformation, transport, decomposition, and discharge of lipids [24]. FTZ also increases the body insulin sensitivity and improves insulin resistance and glucose intake in HepG2 cells [22]. FTZ has been demonstrated to have good therapeutic effects for some diseases with insulin resistance as the central pathogenesis, such as hyperlipemia, nonalcoholic fatty liver disease [22, 24–27]. However, it is still unclear if FTZ has a protective effect on islet *β* cells. In this paper, we explored the protective effect of FTZ on islet *β* cells in vivo and in vitro and analyzed its mechanism.

## 2. Materials and Methods

### 2.1. FTZ Preparation

FTZ was prepared by the Institute of Chinese Medicine, GDPU. The preparation method is the same as previously reported[24, 27]. Eight comprised crude herbs were purchased from Zhixin Pharmaceutical Ltd., Guangzhou. A voucher specimen was deposited in the Institute of Chinese Medicine of Guangdong Pharmaceutical University.

### 2.2. Experimental Animals

Adult male healthy Sprague-Dawley (SD) rats (weighing 180-220 g) were kept under a 12 h light/dark cycle, controlled temperature (25±1°C), and relative humidity of 40%~60% and had free access to standard lab chow and tap water. All animals were purchased from Guangdong Medicinal Laboratory Animal Center (the experimental animal use license number: SYXK (Guangdong) 2012-0125; animal quality certificate No. 44007200019594). This study was carried out in strict accordance with the recommendations in the Guide for the Care and Use of Laboratory Animals of the National Institutes of Health (NIH publication No. 85-23, 1985). The protocol was approved by the Laboratory Animal Ethics Committee of Guangdong Pharmaceutical University (GDPULAEC No. 201502) (Protocol Number: SPF2012132). The whole surgery was performed under Nembutal anesthesia, and all efforts were made to minimize suffering.

#### 2.2.1. Preparation and UPLC-MS Analysis of FTZ-Containing Serum of Rats

Forty healthy SD male adult rats were equally distributed into two groups. In Group One, each animal was orally administered an FTZ solution at a dose of 3 g (FTZ powder)/kg (after fasting for 8 h) twice a day for three days. Blood was obtained through the abdominal aorta 1 h after the last administration and then centrifuged (3,000 r/min, 15 min/times, twice) after 1 h at room temperature. The serum from this group was called FTZ serum. Group Two rats were orally administered water in the same protocol, and the serum from this group was called rat serum. Both the FTZ serum and the rat serum were inactivated by heating at 56°C for 30 min, then filtered through 0.22 *μ*m filters, and stored at -80°C until use. The FTZ preparation, FTZ serum and rat serum were analyzed with combined UPLC/Q-TOF-MS as Zhong reported previously [27].

### 2.3. In Vivo Experiments

#### 2.3.1. Animal Models and Grouping

To induce hyperlipidemia and hyperglycemia by a high-fat and high-sugar diet (HFHSD), male SD rats were randomly divided into 2 groups after a week of adaptive feeding: control group (fed standard lab chow obtained commercially from the Guangdong Medicinal Laboratory Animal Center, composed of 24.0% protein, 3.5% lipid, and 60.5% carbohydrate, n=11) and model control group (fed an HFHSD composed of 24.0% protein, 10% lipid, 35% sucrose, and 31% carbohydrate, n=44). After 6 weeks of HFHSD feeding and 12 h of fasting, the model rats were given 35 mg/kg of streptozocin (STZ, prepared with a fresh citrate buffer solution (pH 4.5)) by intraperitoneal injection. The control rats were administered 1 ml/kg citrate buffer. The fasting blood glucose was measured 3-7 d later with tail vein blood and a glucose meter every week. The hyperglycemia rat model was determined to be successful with a fasting glucose level of 11.1 mmol/L or higher[21].

To evaluate the protective effects of FTZ on the pancreas beta cell in the HFHSD-induced diabetic rat, the 44 identified hyperglycemic rats were then randomly divided into four groups: model group, rosiglitazone group, low-dose FTZ group, and high-dose FTZ group. Each group had 11 rats (n=11). Before grouping, there was no significant difference in the blood glucose and weight between the control group and the model group. After grouping, the remaining four groups of animals continued to give HFHSD, except for the control group.

The treatment rats were orally administered with rosiglitazone (0.84 mg/kg/d), low-dose FTZ (3 g/kg/d), or high-dose FTZ (6 g/kg/d) by gavage. The vehicle rats were orally dosed with the same volume of 1% sodium carboxymethyl cellulose (CMC-Na) solution, and the identical subsequent treatments lasted continuously for 8 weeks.

#### 2.3.2. Sampling and Bioassays

To detect the oxidative stress in the pancreatic tissue of the HFHSF diabetic rats, the MDA, CAT, and SOD in the pancreatic tissue were assayed.

At the end of the experimental period, following fasting for 12 h, the rats were anesthetized with ip 50 mg/kg B. W. of 3% Nembutal solution. Blood was collected from the abdominal aorta and left at room temperature for coagulation. The serum samples were obtained by centrifugation at 3,000×g and 4°C for 10 min and stored at -70°C for later analysis.

Animals were sacrificed by decapitation under anesthesia, and the peritoneal cavity was flushed immediately with 10 ml of cold saline. The pancreases were excised, quickly chilled in liquid nitrogen and stored at -70°C for later analysis; 0.2 g of the pancreas of each rat was homogenized in prechilled phosphate-buffered saline (PBS, pH 7.2) (0.10 g/ml) at 4°C. The supernatant was then centrifuged at 12,000×g and 4°C for 10 min, adjusted to the indicated concentration, and stored at -70°C for future use. The protein content of the supernatant was detected with the Bradford method using a protein assay kit. The MDA content and the activity of CAT and SOD in the pancreatic tissue were determined by using MDA, CAT, and SOD kits according to the manufacturer's protocol.

#### 2.3.3. Histochemical Analysis

The pancreases were quickly removed and placed in 4% paraformaldehyde in 0.1 M PBS fixative solution at 4°C overnight and then fixed with formalin. Pancreases were embedded in paraffin, followed by preparation of the coronal sections, which were 6 *μ*m thick, using a microtome (Leica RM 2135, Nussloch, Germany). The paraffin-embedded transections were deparaffinized with xylene and dehydrated with ethanol at graded concentrations of 100-70% (v/v), followed by washing with water. The sections were stained with an HE staining kit (GeneCopoeia, Inc., USA) according to the manufacturer's protocol and examined using light microscopy (Axio observer A1 photomicroscope [Carl Zeiss, Germany]). The number of surviving pancreatic cells (Nissl-body-positive cells) per 1mm length was counted as the pancreatic cell density.

### 2.4. In Vitro Experiments

#### 2.4.1. Cell Culture

INS-1 islet *β* cells (INS-1 cell) were cultured in RPMI-1640 complete medium containing 10% FBS, 50 *μ*mol/L *β*-sulfhydryl ethanol, 1 mmol/L pyruvate, 100 U/L penicillin, and 100 mg/L streptomycin in a 37°C, 5% CO_2_ incubator. The medium was refreshed once every 3 days. When the culture reached 100% confluency, the cells were dissociated with 0.02% ethylene diamine tetraacetic acid (EDTA) solution and 0.25% trypsin solution at 37°C for 5 min. The dissociation was terminated with RPMI-1640 complete medium. The suspension was centrifuged at 800 rpm for 5 min, and then the cells were resuspended in RPMI-1640 complete medium. The cells were diluted with RPMI-1640 complete medium to approximately 2×10^5^/L, plated onto 6-well-plates, and then incubated at 37°C, 5% CO_2_, and 95% humidity for 48 h. After the culture reached 80% confluence, the medium was discarded, and cells were maintained in RPMI-1640 complete medium with different concentrations of glucose and FTZ serum. High-glucose induction of oxidative stress was conducted [28].

#### 2.4.2. High-Glucose-Induced Oxidative Stress Model and Cell Culture Treatment

For individual experiments, INS-1 cells were divided into a control group (cultured in RPMI-1640 medium with 11 mmol/L glucose and 10% FBS), a model group (cultured in RPMI-1640 medium with 25 mmol/L glucose (high-glucose, HG) and 10% FBS), an edaravone group (cultured in RPMI-1640 medium with HG, 10% FBS, and edaravone with a concentration of 200 *μ*mol/L), a 10% rat serum group (cultured in RPMI-1640 medium with HG and 10% rat serum), 0.4% FTZ serum group (cultured in RPMI-1640 medium with HG, 0.4% FTZ serum, and 9.6% rat serum), 2% FTZ serum group (cultured in RPMI-1640 medium with HG, 2% FTZ serum, and 8% rat serum), and a 10% FTZ serum group (cultured in RPMI-1640 medium with HG and 10% FTZ serum). All groups were cultured for 48 h [28]. The cultures were washed with ice-cold phosphate-buffered saline (PBS) and harvested for the detection of cell viability, apoptosis, ROS, SOD, MDA, Bcl-2, Bax, caspase-3, and caspase-9.

#### 2.4.3. Cell Viability Assay

Cell viability, an indication of the cytotoxicity of FTZ and HG, was evaluated using a CCK-8 assay kit, and the absorbance at 570 nm was measured with a microplate reader according to the CCK-8 manufacturer's instruction [28, 29].

#### 2.4.4. INS-1 Cell Apoptosis Assays

The protective effects of FTZ on INS-1 islet *β* cells induced by HG were measured by determining the number of apoptotic cells as described by Ho [30]. INS-1 cells were plated into 12-well plates and treated with HG and FTZ. Annexin V/FITC and propidium iodide double staining were used to evaluate the percentages of apoptosis. The apoptosis ratio was analyzed after all treatments via using Annexin V/FITC Apoptosis Detection Kit (BD Biosciences, San Diego, CA) according to the manufacturer's instructions.

#### 2.4.5. Measurement of MDA in INS-1 Cell Culture

To assess the antioxidants and lipid peroxides in INS-1 cells after an HG insult, the cultures were harvested, washed with ice-cold PBS, then pooled in 0.1 M PBS/0.05 mM EDTA buffered solution, and homogenized. The homogenate was centrifuged for 1 h at 10, 000* g* at 4°C. The supernatants were used in the assay. We assessed the content of protein in cells by the Bradford method using a protein assay kit. The content of intracellular MDA was determined colorimetrically with a commercial assay kit for MDA (Nanjing Jian Chen BioChem) following the manufacturer's instructions.

#### 2.4.6. Determination of ROS Level

To explore if FTZ-containing serum INS could improve oxidative stress injury caused by a high-glucose insult in ISN-1 cells, intracellular ROS levels were determined by a fluorescence probe dichlorofluorescein diacetate (DCFH-DA) assay with a Reactive Oxygen Species Assay Kit (Beyotime Company, Haimen, China) through inversion fluorescence microscope observation as Gomes described [31]. Briefly, cells (4×10^4^ cells/ml) were seeded into 96-well plates and 2 days later were pretreated with FTZ for 24 h prior to exposure to HG. After the indicated treatments, INS-1 cells cultured in 96-well plates were incubated in the dark with 10 *μ*M DCFH-DA for 30 min at 37°C and were washed twice with PBS. Fluorescence emission at 525 nm from 488 nm excitation was measured on a fluorescence microplate reader (BERTHOD Technologies, Mithras LB 940) and expressed as a percentage of the DCF fluorescence generated in control cells under identical incubation conditions [31].

#### 2.4.7. Western Blot Assay

To analyze the expression of Mn-SOD, CAT, Bcl-2, Bcl-xl, and Bax of the pancreatic tissue and the INS-1 cell at the protein level, a Western blot analysis was conducted; 0.1 g of the pancreatic tissue of different rats or the collected cultured ISN-1 cells of different treatment groups were lysed in 200 *μ*L of RIPA for 30 min on ice and centrifuged at 12, 000 g and 4°C for 10 min. The supernatant was collected and stored for Western blot analysis.

A total of 20 *μ*g of protein from each sample was separated by 8% sodium dodecyl sulfate polyacrylamide gel electrophoresis and electroblotted onto a polyvinylidene difluoride membrane (GE Healthcare, Buckinghamshire, UK) using a Hoefer semidry blotter. The membrane was blocked for 2 h at room temperature in 5% nonfat dried milk/Tris-buffered saline containing Tween 20 and incubated with the indicated antibody (Mn-SOD, 1:1000; CAT, 1:1000; Bcl-2, 1:1000; BCl-xl, 1:1000; Bax, 1:1000; and *β*-actin, 1:1000) at 4°C overnight. After washing three times with TBS-T for 10 min each time, the membrane was incubated with horseradish peroxidase-conjugated goat anti-rabbit secondary antibody (Abcam, 1:5000) for 2 h at room temperature, washed three times as described above, and visualized using an enhanced chemiluminescence kit (B&M Innovation, Oak Hill, FL, USA). The blot signal on the film was detected and quantified using Image Master VDS (SYNERGY Gene Company Limited) with an image analysis software (Image Master Total Lab; SYNERGY) [24, 32].

#### 2.4.8. Determination of Caspase-3, -9 Activity in INS-1 Cells by ELISA Assays

The activity of caspase-3 and caspase-9 was determined using the caspase-3/-9 activity kit (Beyotime Institute of Biotechnology, Haimen, China) based on a colorimetric assay of the yellow formazan chromophore p-nitroaniline (pNA) after cleavage from the labeled substrate DEVD-pNA or LEHD-pNA. To evaluate the activity of caspase-3 or caspase-9, cells were homogenized in 80/100*μ*l reaction buffer (1% NP-40, 20 mM Tris-HCl (pH 7.5), 137 mM NaCl, and 10% glycerol) containing 10 *μ*l caspase-3 substrate (Ac-DEVD-pNA, 2 mM) or caspase-9 substrate (Ac-LEHD-pNA, 2 mM) after all treatments. Lysates were incubated at 37°C for 2 h. Samples were measured with an ELISA reader (Multiskan Ascent) at an absorbance of 405 nm. The detailed analysis procedure is described in the ELISA manufacturer's protocol. The caspase activity, normalized for total proteins of cell lysates, was then expressed as fold of the baseline caspase activity of control INS-1 cells.

### 2.5. Statistical Analysis

Raw data were analyzed with SPSS 13.0 and GraphPad Prism 5.0 software (GraphPad Software, Inc., San Diego, CA).

All results were expressed as the mean ± SD. The data were evaluated by one-way ANOVA, and the differences between the means were assessed using Duncan's test; p<0.05 was considered statistically significant.

## 3. Results

### 3.1. UPLC-ESI-MS Fingerprint of FTZ Extracts and FTZ Serum

**Figure 1:**
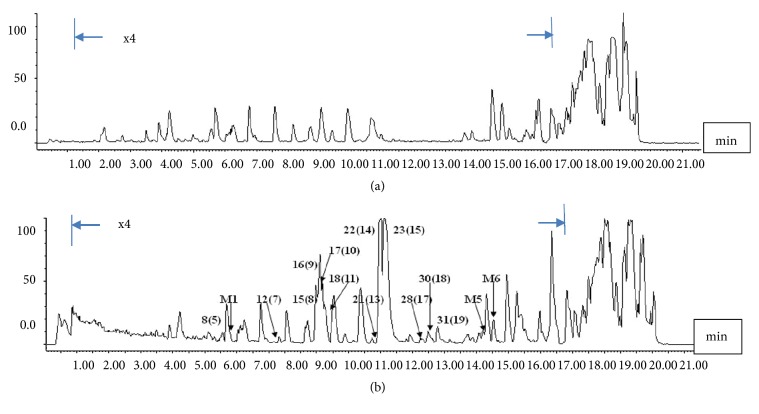
Total ion flow diagram of FTZ. FTZ serum by UPLC-ESI-MS analysis. The blood of the rats was collected from the control group or the 3 g/kg FTZ group after the FTZ, or vehicle was administered for 1 h. The samples were analyzed with UPLC-ESI-MS after the blood sample had been prepared as the text presented. The total ion flow diagram of control and FTZ serum by UPLC-ESI-MS analysis was obtained.

The total ion flow diagram and the positive ion mode diagram by UPLC-ESI-MS of FTZ and rat FTZ serum were obtained as shown in Figure 1. The FTZ serum atlas was compared with the FTZ spectrum, and the UPLC-ESI-MS total ion flow diagram of FTZ-containing serum was roughly in line with the FTZ original peak form. The MS^+^ (m/z) shown in the FTZ serum atlas is roughly the same as the MS^+^ (m/z) shown in the FTZ extracts. It can be seen that the FTZ serum contained a certain concentration of FTZ component content, such as berberine, coptisine, palmatine, notoginsenoside R1, ginsenoside Rg1, ginsenoside Rb1, ginsenoside Rd, ginsenoside Rh1, ginsenoside F1, protopanaxatriol, thalifendine, hydroxyl-palmatine, columbamine, epiberberine, jatrorrhizine, coniferin, maslinic acid, pomolic acid acetate, oleanolic acid, protocatechuic acid, eucommiol, and 5,7-dimethoxycoumarin. In total, more than 27 prototype constituents were identified from UPLC-MS in the FTZ serum. This finding revealed that most of the alkaloids, ginsenosides, and pentacyclic triterpenes could be unambiguously detected in their original forms from the rat serum after FTZ was administered (Supplement Table 2). The results of rat FTZ serum and FTZ extracts by UPLC-ESI-MS were as previously presented [27].

### 3.2. Effect of FTZ on Protection of *β* Cells in the Pancreas of the HFHSD Diabetic Rats

**Figure 2:**
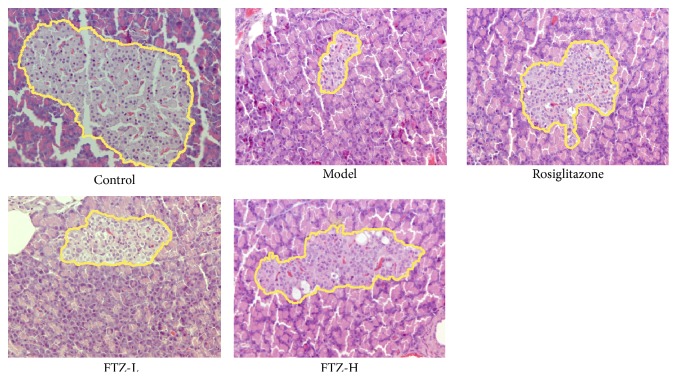
Effect of FTZ on protection of pancreatic islets of the HFHSD diabetic rats. The level of pancreatic islets in HFHSD diabetic rats was assayed as described in the text. Rats were fed with HFHSD for 16 weeks and injected with 36 mg/kg STZ* ip* at the end of the 6th week. FTZ and rosiglitazone were administered for 8 weeks. The pancreatic islet cell levels of different rats were assayed with an HE staining microphotograph (400×).

HFHSD-fed rats showed obvious hyperglycemia. The numbers of pancreatic *β* cells of the HFHSD-fed diabetic rats were significantly decreased in the area and quantity count compared with the control rats. FTZ and rosiglitazone increased the area and cell quantity of the pancreatic *β* cells of HFHSD-fed model rats (Figure 2).

### 3.3. Effect of FTZ on MDA, SOD, and CAT in the Pancreatic Tissue of the HFHSD Diabetic Rats

As shown in Table 1, MDA levels were higher in the HFHSD diabetic model rats than in the control group, and the levels of SOD and CAT were significantly lower in the HFHSD diabetic model rats than in the control group (p < 0.01). FTZ increased the SOD and CAT activity and reduced the MDA contents in the pancreatic tissue of HFHSD-fed model rats (p<0.05,0.01) in a dose-dependent way, and rosiglitazone showed a similar result (Table 1).

### 3.4. Effect of FTZ on the Protein Expression of Bcl-2 and Caspase-3 in the Pancreatic Tissue of the HFHSD Diabetic Rats

**Figure 3:**
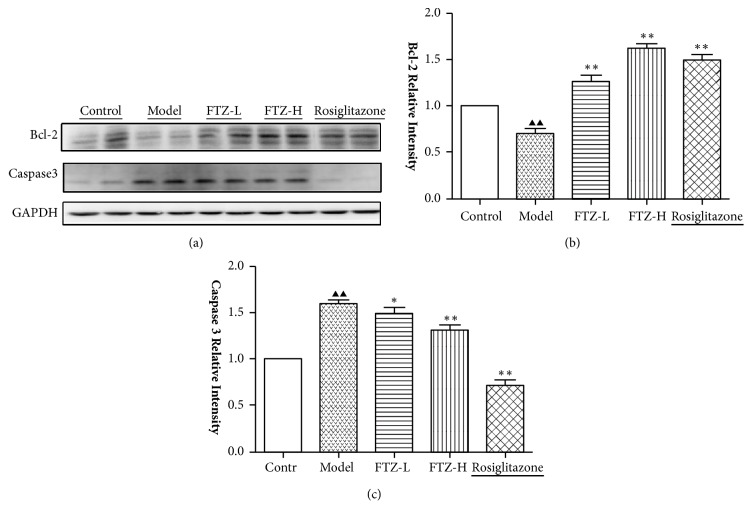
Effect of FTZ on the protein expression of Bcl-2 and caspase-3 in the pancreatic tissue of the HFHSD diabetic rats. The levels of protein expression of Bcl-2 and Caspase-3 in pancreatic islets of HFHSD diabetic rats were assayed as described in the text. Rats were fed with HFHSD for 16 weeks and injected with 36 mg/kg STZ* ip* at the end of the 6th week. FTZ and rosiglitazone were administered for 8 weeks. The protein expression of Bcl-2 and caspase-3 in the pancreatic tissue of the HFHSD diabetic rats was tested by Western blot (a). The protein expression relative intensity of Bcl-2 (b) and caspase-3 (c). Compared with control group^▲▲^P<0.01. Compared with model group, *∗*P<0.05; *∗∗*P<0.01.

As shown in Figure 3, in the HFHSD diabetic model rats, caspase-3 levels were higher and Bcl-2 levels were significantly lower than those in the control group (p < 0.01). FTZ increased the Bcl-2 level and reduced the caspase-3 expression in the pancreatic tissue of HFHSD-fed model rats (p<0.05,0.01) in a dose-dependent way, and Rosiglitazone also showed a similar result (p<0.05)(Figure 3).

### 3.5. Effect of FTZ-Containing Serum on the Viability of *β* Cells

**Figure 4:**
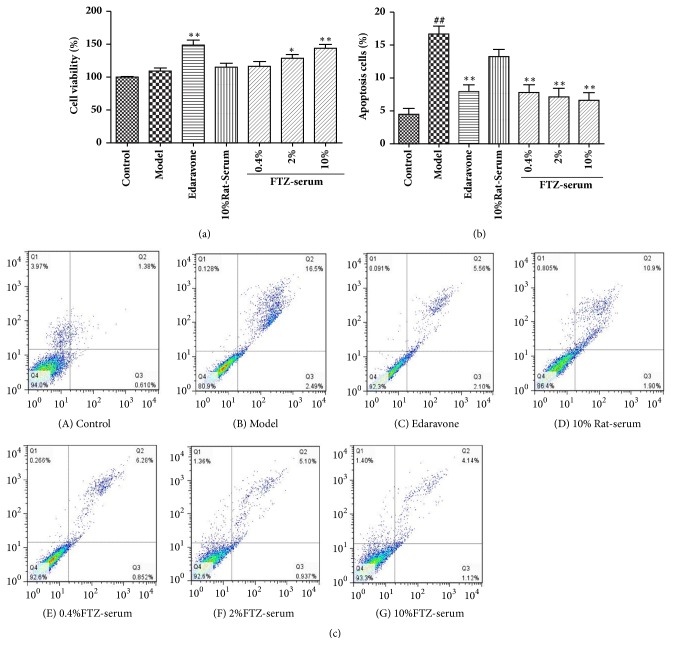
Effects of FTZ serum on high-glucose (25 mM)-induced cytotoxicity in INS-1 cells. INS-1 cells were treated with different concentrations of glucose and different concentrations of FTZ serum for 24 h. Cell viability was assessed using the CCK-8 assay kit. Cell viability was measured to calculate the percentage of untreated INS-1 cells (a). Apoptosis rate by flow cytometry with Annexin V-FITC/PI double staining (b, c) after INS-1 cells were treated with control (11 mM glucose +vehicle); (B) Model (25 mM glucose +vehicle); (C) 200 *μ*M edaravone, (D) 10% rat serum, (E) 0.4% FTZ serum, (F) 2. 0% FTZ serum, and (G) 10% FTZ serum for 24 h before incubation with 25 mM glucose for 24 h. n=6 in each group. Compared with the control group, ^#^P<0.05; ^##^P < 0.01. Compared with model group, *∗*P<0.05; *∗∗*P<0.01.

Compared with the control group, the high concentration of glucose showed a mildly increasing effect on the proliferation rate of INS-1 cells, but there was no significant difference. Compared with model group cells, the free radical scavenger edaravone showed a significant increasing effect on the proliferation of the INS-1 cells (P< 0.01). The serum of the rats and the serum of different concentrations of FTZ had a catalytic effect on the proliferation of *β* cells. The 2% FTZ-containing serum and 10% FTZ-containing serum showed a significant increasing effect on the cell viability concentration dependently (P< 0.05, P< 0.01 respectively, Figure 4(a)).

### 3.6. Effect of FTZ-Containing Serum on Apoptosis of INS-1 Cells under Oxidative Stress

The apoptosis detecting result was shown in Figure 4. It could be seen from the INS-1 islet *β* cell culture microphotograph that the control group cells had great transparency, a strong refractive index, and an unclear contour, with clear tiny cell structures and a lower percentage of apoptotic cells. Compared with the control group, the high-glucose-induced oxidative stress model cell showed weaker refraction, clearer contours, cytoplasmic vacuoles and particle material, an increased gap between cells, and an irregular shape, and some cells even floated. The cell apoptosis rate was significantly increased (p< 0.01) by 72.4%. Compared with model group cells, the cells in edaravone, 0.4%, 2%, and 10% of the FTZ-containing serum group were in better condition with fewer floating cells, closely linked cells, a clear tiny cell structure, and a significantly decreasing apoptosis rate (p< 0.01). In addition, 0.4%, 2%, and 10% of the FTZ-containing serum had significantly inhibited INS-1 cell apoptosis caused by oxidative stress (p< 0.01), and the inhibition rates were 52.51%, 56.27%, and 59.27%, respectively. However, the cell status of the blank serum group was not improved, and the cell apoptosis rate in this group was even higher than that of the model group (Figure 4(c)).

### 3.7. Effect of FTZ-Containing Serum on the SOD and MDA Levels in INS-1 Cells Exposed to HG

**Figure 5:**
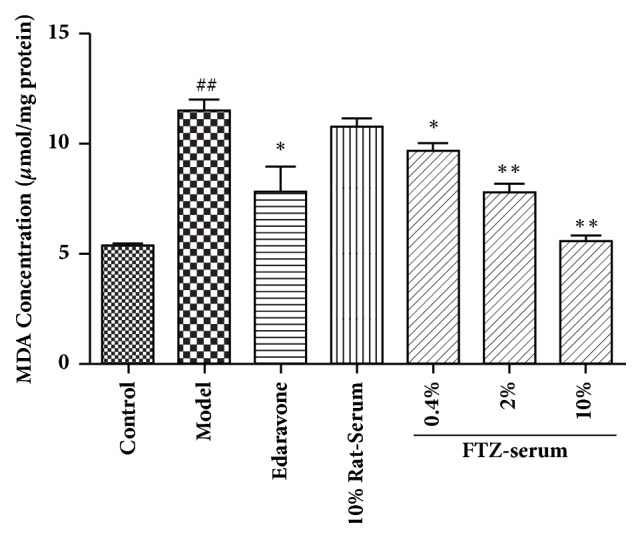
Effect of FTZ-containing serum on the level of MDA in INS-1 cells under 25 mM glucose-induced oxidative stress. INS-1 cells were treated as described in the text. The intracellular MDA was assayed with an MDA assay kit. n=6 in each group. Compared with the control group, ^##^P < 0.01. Compared with model group, *∗*P<0.05; *∗∗*P<0.01.

As shown in Figures 5 and 8(a), HG-induced SOD reduction and MDA increased by 54.16% in the INS-1 cells exposed to HG. Pretreatment with FTZ-containing serum significantly enhanced the intracellular SOD (Figure 8(a)) and reduced the intracellular MDA content (Figure 5) in the INS-1 cells exposed to HG in a dose-dependent manner. A similar effect was observed with edaravone (Figures 5 and 8(a))

### 3.8. Effect of FTZ-Containing Serum on ROS in HG-Insulted *β* Cells

**Figure 6:**
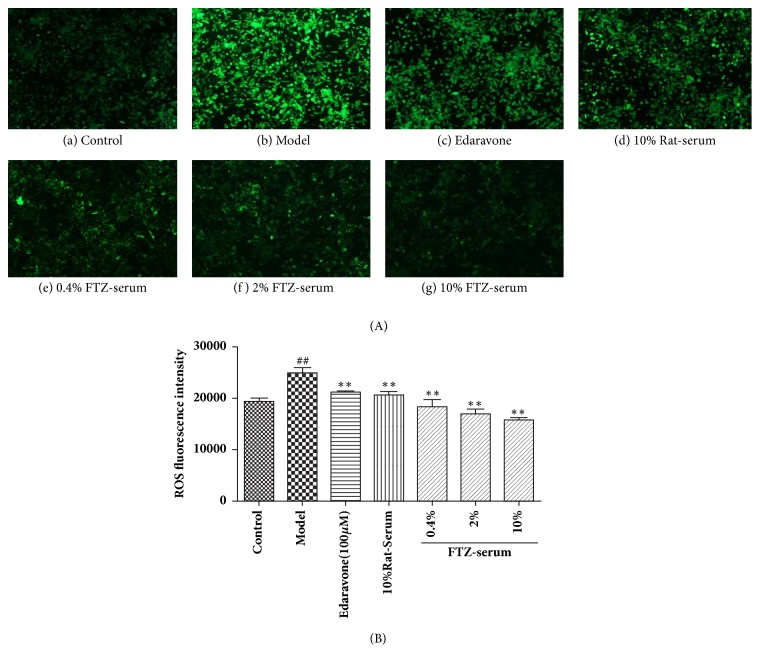
Effect of FTZ serum on the level of ROS in INS-1 cells under 25 mM glucose-induced oxidative stress. Cells were untreated or pretreated with different concentrations of FTZ serum or edaravone for 24 h before incubation with 25mM glucose (HG) for 24 h. (A) Fluorescence (DCF)-labeled cells in (a) untreated control; (b) HG^+^ + 0.1% DMSO; (c) HG^+^ + 30 *μ*M edaravone; (d) HG+ 10% rat serum; (e) HG+ 0.4% FTZ serum; (f) HG+ 2% FTZ serum; and (g) HG^+^ + 10% FTZ serum were observed under a microscope with a scale bar of 100 *μ*m. (B) The ROS fluorescence intensity (Intracellular ROS generation) in each group was calculated by the rate of untreated naïve INS-1 cells. Values represent the mean ± SD in 3 independent experiments. Compared with the control group, ^##^P < 0.01. Compared with model group, *∗*P<0.05; *∗∗*P<0.01.

The results in Figure 6 show that high-glucose culture induced a significant increase in the intracellular ROS in the INS-1 cells, as indicated by the green fluorescence intensity (P < 0.01) in the model group compared with the control group(P < 0.01, Figures 6(a) and 6(b)). Pretreatment with FTZ significantly decreased the intracellular ROS in a concentration-dependent manner, and edaravone also showed a similar effect on INS-1 cells ROS (Figures 6(c) and 6(e)–6(g)). The ROS fluorescence intensity decreased significantly (P < 0.01) in the edaravone group, 10% rat serum group, and 0.4% FTZ serum group (P < 0.01), with a decrease of 14.8%, 20.2%, and 26.3%, respectively. The decrease in ROS fluorescence intensity in the 2.0% FTZ serum and 10% FTZ serum cells was more significant (P < 0.01) with a decrease of 31.7% and 37.2%, respectively. The fluorescence intensity of the ROS was significantly decreased in 2% and 10% of FTZ serum cells compared with 10% rat serum group cells (P < 0.01), and the decrease was 22.19% and 24.4% respectively.

### 3.9. Effect of FTZ-Containing Serum on Caspase-3, -9 Activity in the INS-1 Cells Exposed to HG

**Figure 7:**
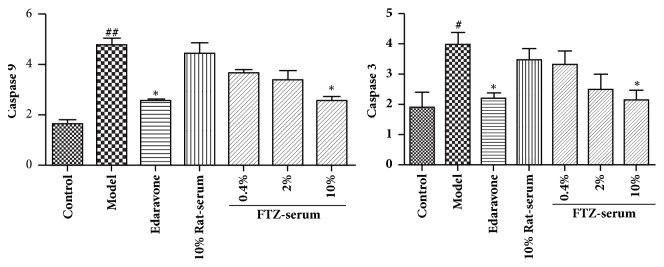
Effect of FTZ serum on the caspase-3, -9 activity in INS-1 cells under high-glucose-induced oxidative stress. INS-1 cells were untreated or pretreated with different concentrations of FTZ serum or edaravone for 24 h before incubation with 25 mM glucose (HG) for 24 h. The activity of INS-1 cell apoptosis protein caspase-9 (A) and caspase-3 (B) was assayed by an ELISA kit. n=5 in each group. Compared with the control group, ^#^P<0.05; ^##^P < 0.01. Compared with model group, *∗*P<0.05; *∗∗*P<0.01.

The results showed that, compared with control group, the activity of caspase-9 and caspase-3 in INS-1 cells of the model group exposed to HG increased significantly, by 65.44% and 48.81%, respectively (P < 0.01). Treatment with FTZ serum significantly decreased the activity of caspase-3, -9 in the INS-1 cells exposed to HG in a concentration-dependent manner, and edaravone treatment also decreased the activity of caspase-9 and caspase-3 in the INS-1 cells under oxidative stress (Figure 7). Compared with blank serum, 10% of FTZ-containing serum significantly reduced the activity of caspase-9 in cells (P < 0.05) by 46.2%. Compared with the model group, 10% of FTZ-containing serum could significantly reduce the activity of caspase-3 in the cells by 46.86% (P < 0.05). However, there was no significant improvement in the activity of caspase-3 and caspase-9 in the INS-1 cells under oxidative stress treated with control serum.

### 3.10. Effect of FTZ-Containing Serum on the Protein Expression of Mn-SOD and CAT in INS-1 Cells Exposed to HG

**Figure 8:**
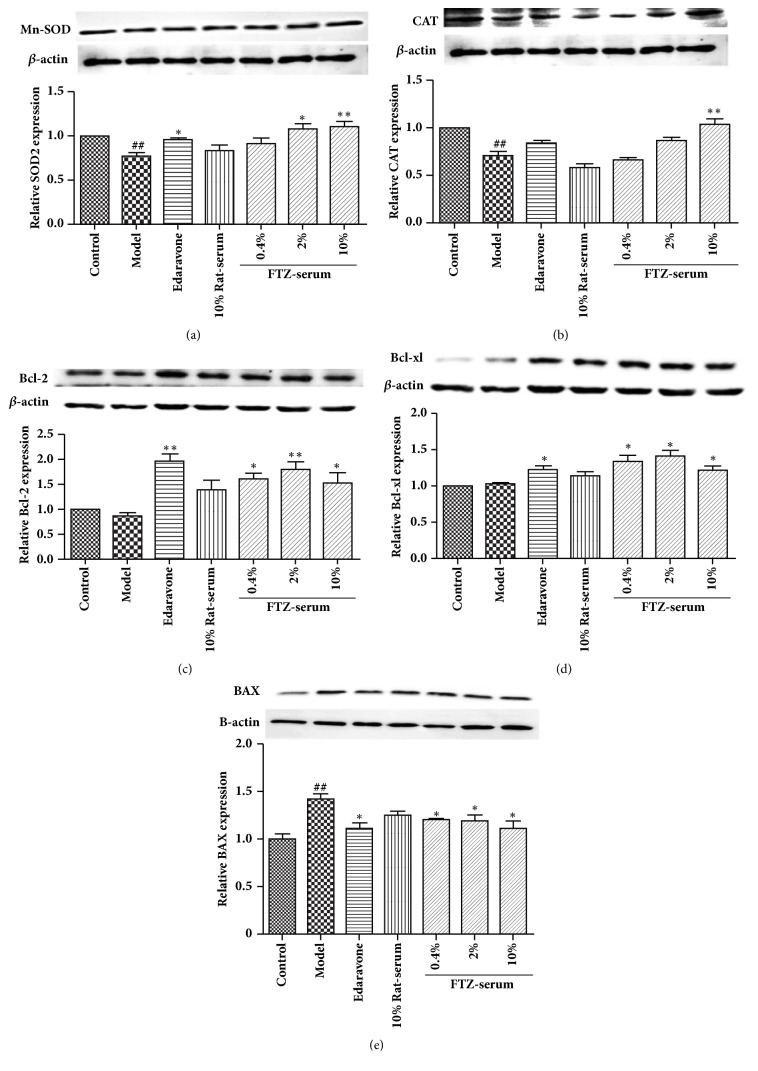
Effect of FTZ serum on the Mn-SOD and CAT as well as Bcl-2, Bcl-xl, and Bax protein expression in INS-1 cells under HG-induced oxidative stress. INS-1 cells were untreated or pretreated with different concentrations of FTZ serum or edaravone for 24 h before incubation with 25 mM glucose (HG) for 24 h. The intracellular SOD (a), CAT (b), Bcl-2 (c), Bcl-xl (d), and Bax (e) protein expression in the INS-1 cells under oxidative stress induced by HG were tested by Western blot. n=6. Compared to the control group, ^#^P<0.05; compared to the model group, *∗*P<0.05.

As the Western blot result shows in Figure 8, compared with the control group, the model group had significantly decreased protein expression levels of Mn-SOD and CAT (P < 0.01). Compared with the cells of the model group, 2% and 10% FTZ-containing serum remarkably increased the Mn-SOD expression in a concentration-dependent manner, by 26.57% and 29.6%, respectively (p<0.05 or 0.01), and the cells of the edaravone group showed increased protein expression levels of Mn-SOD with a significant difference (p< 0.05)(Figure 8(a)).

According to the result in Figure 8(b), compared with control group, the model group showed a significantly decreased expression level of CAT (P < 0.01). Compared with the model group, the 10% FTZ serum group showed a significantly increased expression level of CAT protein (P < 0.01), and there was no significant change in the expression level of CAT in the edaravone-treated and the 0.4% and 2% FTZ serum-treated cells.

### 3.11. Effect of the FTZ-Containing Serum on the Expression of Bcl-2 Family Proteins in the INS-1 Cells Exposed to HG

The Western blot results showed that there was no significant change in the expression levels of Bcl-2 and Bcl-xl proteins in the model group compared to the control group. Compared with the model group, in the edaravone group, the expression level of Bcl-2 and Bcl-xl protein was significantly increased (p < 0.01). However, 0.4%, 2%, and 10% of FTZ-containing serum significantly increased the expression of Bcl-2 protein in the cells by 43.74%, 49.68%, and 39.71%, respectively (Figure 8(c)).

In addition, 0.4%, 2%, and 10% of FTZ-containing serum significantly increased the expression of Bcl-xl protein in the cells by 21.48%, 26.49%, and 14.93%, respectively (Figure 8(d)).

WB results also showed that the expression of Bax protein in the model group was significantly increased by 29.62% (p< 0.01). Compared with model group cells, 0.4%, 2%, and 10% of FTZ-containing serum significantly reduced the expression of Bax protein in the INS-1 cells exposed to HG, by 14.84%, 15.8%, and 21.28%, respectively. Edaravone-treated cells showed a significantly reduced expression of Bax protein (p< 0.05). In addition, the effect of rat blank serum on Bax protein expression was not significant (Figure 8(e)).

## 4. Discussion

Our study showed that FTZ protected pancreatic *β* cells from HFHSD-induced damage by improving SOD, CAT, and Bcl-2 and reducing the level of Caspase-3 and MDA in rats fed a HFHSD. We also demonstrated that FTZ protected pancreatic *β* cells from damage from exposure to high concentrations of glucose by reducing ROS and MDA and increasing Mn-SOD and CAT. The findings provide fundamental support for FTZ use in the clinical treatment of type 2 diabetes.

Type 2 diabetes is essentially an oxidation-inducing disease [1, 3]. ROS located in the cytoplasm could lower the expression of Bcl-2 protein in the mitochondrial membrane and increase the expression of apoptotic proteins Bad and Bak, causing subsequent amplification of the cell apoptosis signaling cascade. Excessive accumulation of mitochondrial ROS may induce mitochondrial DNA mutations, damage mitochondrial membrane phospholipids, open the mitochondrial permeability transition pore (MPTP) and lining anion channel, lead to more ROS release into the cytoplasm, increase oxidative stress damage, and, finally, lead to cell apoptosis [4–6, 32].

Caspase-3 and caspase-9, which further activate apoptosis proteins, are key cell apoptosis proteins in the apoptosis signaling pathway [5, 7].

Oxidative stress is one of the mechanisms of glucose toxicity in *β* cell damage in the pancreatic islet [9]. Long-term high blood glucose levels might make *β* cells produce excessive ROS, which could damage cells through different ways including glycosylation end product formation, DNA damage, and multiple polymerase activation, eventually leading to *β* cell function loss, even *β* cell apoptosis [3–5].

Our result showed that diabetes was successfully induced by feeding with HFHGD and combined with streptozotocin intraperitoneal injection in rats, and the *β* cells in the pancreases of the diabetic rats were damaged, accompanied by a higher content of MDA, higher caspase-3 activity, and lower SOD, CAT, and Bcl-2 levels in the pancreatic tissue, which confirmed that the pancreases of the rats were insulted by oxidative stress.

Furthermore, the oxidatively stressed *β* cell model was successfully induced by using RPMI-1640 medium containing 25 mmol/L glucose to culture the INS-1 *β* cell line, which was characterized by an increased accumulation of ROS and lipid peroxide MDA and reduced Mn-SOD and CAT protein expression significantly in INS-1 *β* cells. Moreover, the expression of antiapoptotic protein Bcl-2/Bcl-xl and apoptotic protein Bax was unbalanced, and the activity of caspase-9 and caspase-3 and the apoptosis rate of cells also increased significantly [29, 31], which caused oxidative stress in *β* cells and finally led to the apoptosis of INS-1 cells. This result is consistent with reports that oxidative stress is one of the key mechanisms in diabetic pathophysiology [3–12].

ROS can damage *β* cells by oxidative stress, lead to a decreased number of *β* cells and insulin secretion in the pancreas, and later induce insulin resistance, eventually causing or aggravating diabetes [9–12]. Therefore, protecting *β* cells, maintaining the number of *β* cells, and ensuring the normal function of *β* cells in the treatment of type 2 diabetes have become increasingly valued [1, 13, 14, 21].

Moreover, the experimental results showed that the expression of Mn-SOD and CAT in *β* cells was improved by different concentrations of FTZ serum, reducing the concentration of ROS and MDA in the cells, and improving the degree of oxidative stress. In addition, the imbalance between the antiapoptotic protein Bcl-2/Bcl-xl and the apoptotic protein Bax induced by oxidative stress was improved by FTZ serum, and the activity of apoptotic protease caspase-9 and caspase-3 was decreased; thereafter, FTZ also reduced the apoptosis rate of the *β* cells.

Activation of caspase-9 in cells can further activate apoptotic protease caspase-3, which results in apoptosis [15–17, 33]. It was found that FTZ inhibited the activity of caspase-3, which might be related to the inhibition of INS-1 cells apoptosis induced by 25 mM glucose.

It was reported that berberine, ginsenosides, oleic acid, and other polyphenols are effective in cleaning up oxidative ROS [21, 22, 24–27]. FTZ is composed of eight traditional Chinese medicinal herbs, such as Buddhist, Huanglian, Nvzhenzi, and Sanqi [24]. UPLC-MS assays showed that FTZ contains berberine, ginsenosides, oleic acid, and other polyphenol ingredients, which are reported to exhibit antioxidative characteristics [24–27, 29]. This might account for the antioxidative stress effect of FTZ. For the development of FTZ as a new Chinese medicine agent, we will further elucidate if berberine, ginsenosides, oleic acid, and other polyphenol ingredients contribute to the protection of *β* cell by improving the oxidative stress in the future.

## 5. Conclusion

Diabetes with significantly higher levels of blood glucose was induced in healthy adult SD male rats by feeding a HFHSD for 6 weeks and then by intraperitoneal injection with 35 mg/kg STZ. FTZ administration for 8 weeks significantly protected the pancreas beta cells and relieved the pancreas damage induced by HFHSD feeding in rats, by increasing the islet *β* area and the islet cell number of the pancreas by reducing oxidative stress in HFHSD diabetic rats.

FTZ-containing serum could protect INS-1 islet *β* cells from high-glucose induced damage, inhibit the apoptosis of islet *β* cells, and improve the insulin release function of islet *β* cells. The mechanism may be related to increased expression of the intracellular antioxidant proteins Mn-SOD and CAT and reduced concentrations of MDA and ROS by improving the different levels of oxidative stress, which may be related to increasing the ability of the antioxidative stress and the expression of the antiapoptotic proteins Bcl-2 and Bcl-xl, reducing the expression of preapoptotic protein Bax in cellular mitochondrial membranes, promoting the balance between apoptotic proteins, and decreasing the activity of apoptotic caspase-9 and caspase-3.

FTZ protected the pancreatic *β* cells by improving oxidative stress, which lays a solid foundation for the clinic application of FTZ in the prevention and treatment of glucolipid metabolic disorders.

## Figures and Tables

**Table 1:** Effect of FTZ on MDA, SOD, and CAT in the pancreas tissue of the HFHSD diabetic rats.

Group	Control	Model	FTZ (3 g/kg)	FTZ (6 g/kg)	Rosiglitazone (0.84 mg/kg)
MDA(nmol/mg pro)	3.06+0.61	5.86±0.98^▲▲^	4.35±1.12^*∗∗*^	3.86±1.38^*∗∗*^	4.02±1.21^*∗*^
SOD (U/mg pro)	25.86±7.35	12.28±3.71^▲▲^	18.61±4.68^*∗*^	21.53±6.91^*∗∗*^	18.25±4.68^*∗*^
CAT (U/g pro)	560.31±70.34	361.36±61.06^▲▲^	431.68±71.31^*∗∗*^	466.10±81.52^*∗∗*^	428.64±60.18^*∗*^

MDA (nmol/mg protein), SOD (U/mg protein), and CAT (U/g protein) in the pancreas tissue of the HFHSD diabetic rats were measured as the Materials and Methods described. Note: n=10 for all groups. Compared with control group, ^▲▲^P<0.01. Compared with model group, *∗*P<0.05; *∗∗*P<0.01.

## Data Availability

The data used to support the findings of this study are included within the article, or within the supplementary information file(s).

## Abbreviations

Bax: Bcl-2 associated X
Bcl-2: B-cell leukemia-lymphoma
Bcl-xl: B-cell lymphoma/leukemia-xl gene
CAT: Catalase
CMC-Na: Carboxymethyl cellulose
DCFH-DA: Dichloro-dihydro-fluorescein diacetate
DMEM: Dulbecco's modified eagle medium
DMSO: Dimethyl sulfoxide
EDTA: Ethylene diamine tetraacetic acid
ELISA: Enzyme linked immunosorbent assay
FTZ: Fufang Zhenshu Tiao-Zhi
HFHSD: High-fat high-sugar diet
HG: High-glucose
InsRs: Insulin receptors
IRS: Insulin receptor substrate
MDA: Malondialdehyde
Mn-SOD: Mn-superoxide dismutase
NF-kB: Nuclear factor-k-gene binding
PBS: Phosphate-buffered saline
pNA: p-Nitroaniline
ROS: Reactive oxygen species
SOD: Superoxide dismutase
STZ: Streptozocin
TCMs: Traditional Chinese Medicine
UPLC-MS: Ultrahigh performance liquid chromatography-mass spectrum
UPLC/Q-TOF-MS: Ultrahigh performance liquid chromatography/mass spectrometry.

## Supplementary Materials

Supplementary Materials Supplement Figure 1: A.* The total ion flow diagram of FTZ, FTZ serum, and control serum by UPLC-ESI-MS analysis. *The blood of the rats was collected from the control group and the FTZ 3 g/kg group after the FTZ or vehicle was administrated for 1 h. The samples were analyzed with UPLC-ESI-MS after the blood sample has been prepared as the text presented. The total ion flow diagram of FTZ (a) FTZ serum (b), and control serum (c) by UPLC-ESI-MS analysis was obtained.* B. The total ion flow diagram at the negative ion model of control (a) and FTZ serum (b) by UPLC-ESI-MS analysis. *The blood of the rats was collected from the control group and the FTZ 3 g/kg group after the FTZ or vehicle was administrated for 1 h. The samples were analyzed with UPLC-ESI-MS after the blood sample has been prepared as the text presented. The total ion flow diagram of control and FTZ serum by UPLC-ESI-MS analysis was obtained.* C. The total ion flow diagram at the negative ion model (a) and the positive ion model (b) of FTZ by UPLC-ESI-MS analysis. *The FTZ samples were analyzed with UPLC-ESI-MS as the text presented. The total ion flow diagram of FTZ by UPLC-ESI-MS analysis was obtained.* Supplement Tables*. Supplement Table 1: UPLC flow phase gradient elution procedure. Supplement Table 2: FTZ serum constituents.
