# Supplementary material for: Chinese Medicine FTZ Recipe Protects against High-Glucose-Induced Beta Cell Injury through Alleviating Oxidative Stress
Source: Evid Based Complement Alternat Med. 2019 Mar 3;2019:6378786. doi: 10.1155/2019/6378786 (PMC6421024; doi:10.1155/2019/6378786)
Supplement: Supplementary Materials — Supplement Figure 1: A. The total ion flow diagram of FTZ, FTZ serum, and control serum by UPLC-ESI-MS analysis. The blood of the rats was collected from the control group and the FTZ 3 g/kg group after the FTZ or vehicle was administrated for 1 h. The samples were analyzed with UPLC-ESI-MS after the blood sample has been prepared as the text presented. The total ion flow diagram of FTZ (a) FTZ serum (b), and control serum (c) by UPLC-ESI-MS analysis was obtained. B. The total ion flow diagram at the negative ion model of control (a) and FTZ serum (b) by UPLC-ESI-MS analysis. The blood of the rats was collected from the control group and the FTZ 3 g/kg group after the FTZ or vehicle was administrated for 1 h. The samples were analyzed with UPLC-ESI-MS after the blood sample has been prepared as the text presented. The total ion flow diagram of control and FTZ serum by UPLC-ESI-MS analysis was obtained. C. The total ion flow diagram at the negative ion model (a) and the positive ion model (b) of FTZ by UPLC-ESI-MS analysis. The FTZ samples were analyzed with UPLC-ESI-MS as the text presented. The total ion flow diagram of FTZ by UPLC-ESI-MS analysis was obtained. Supplement Tables. Supplement Table 1: UPLC flow phase gradient elution procedure. Supplement Table 2: FTZ serum constituents. [file 6378786.f1.zip › 6378786.f1/Supplement Fig 1_ECAM_2600255.docx]

Supplement Fig 1

A
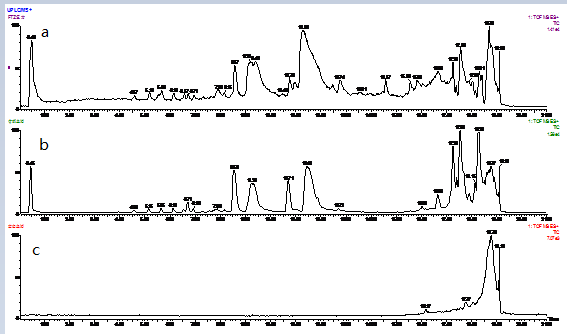


B
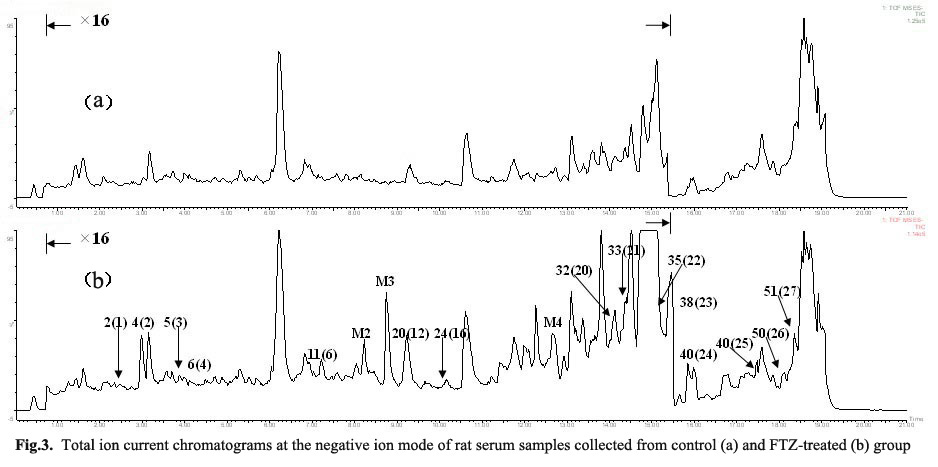


C
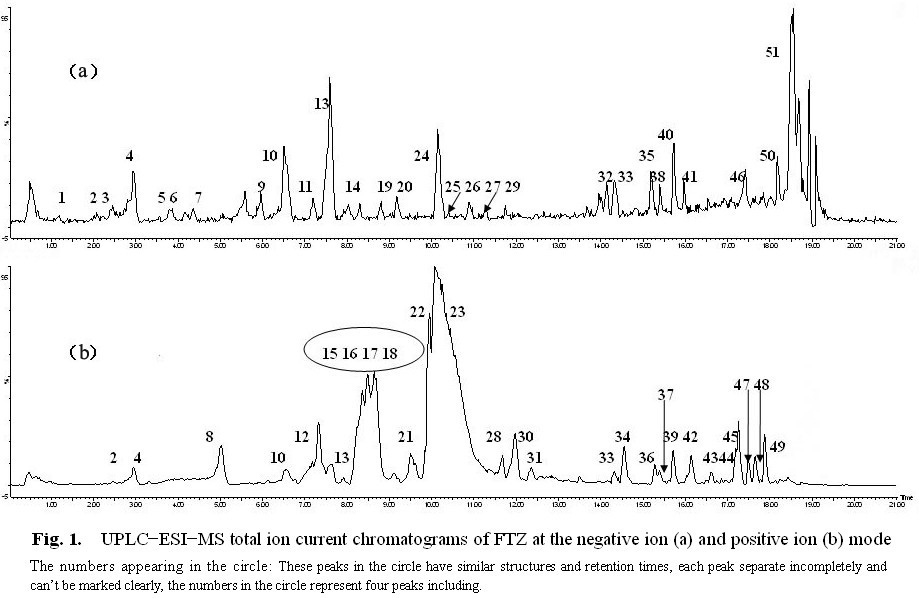


A.**The total ion flow diagram of FTZ, FTZ serum and control serum by UPLC-ESI -MS analysis**

The blood of the rats was collected from the control group, FTZ 3 g/kg group after the FTZ or vehicle was administrated for 1 h. The samples were analyzed with UPLC-ESI-MS after the blood sample has been prepared as the text presented. The total ion flow diagram of FTZ(a) FTZ serum(b) and Control serum (c)by UPLC-ESI -MS analysis was obtained.

**B The total ion flow diagram at the negetive ion model of control(a), FTZ serum(b) by UPLC-ESI -MS analysis**

The blood of the rats was collected from the control group, FTZ 3 g/kg group after the FTZ or vehicle was administrated for 1 h. The samples were analyzed with UPLC-ESI-MS after the blood sample has been prepared as the text presented. The total ion flow diagram of Control and FTZ serum by UPLC-ESI -MS analysis was obtained.

**C. The total ion flow diagram at the negetive ion model (a) and the positive ion model (b) of FTZ by UPLC-ESI -MS analysis**

The FTZ samples were analyzed with UPLC-ESI-MS as the text presented. The total ion flow diagram of FTZ by UPLC-ESI -MS analysis was obtained.
