# Supplementary material for: Chinese Medicine FTZ Recipe Protects against High-Glucose-Induced Beta Cell Injury through Alleviating Oxidative Stress
Source: Evid Based Complement Alternat Med. 2019 Mar 3;2019:6378786. doi: 10.1155/2019/6378786 (PMC6421024; doi:10.1155/2019/6378786)
Supplement: Supplementary Materials — Supplement Figure 1: A. The total ion flow diagram of FTZ, FTZ serum, and control serum by UPLC-ESI-MS analysis. The blood of the rats was collected from the control group and the FTZ 3 g/kg group after the FTZ or vehicle was administrated for 1 h. The samples were analyzed with UPLC-ESI-MS after the blood sample has been prepared as the text presented. The total ion flow diagram of FTZ (a) FTZ serum (b), and control serum (c) by UPLC-ESI-MS analysis was obtained. B. The total ion flow diagram at the negative ion model of control (a) and FTZ serum (b) by UPLC-ESI-MS analysis. The blood of the rats was collected from the control group and the FTZ 3 g/kg group after the FTZ or vehicle was administrated for 1 h. The samples were analyzed with UPLC-ESI-MS after the blood sample has been prepared as the text presented. The total ion flow diagram of control and FTZ serum by UPLC-ESI-MS analysis was obtained. C. The total ion flow diagram at the negative ion model (a) and the positive ion model (b) of FTZ by UPLC-ESI-MS analysis. The FTZ samples were analyzed with UPLC-ESI-MS as the text presented. The total ion flow diagram of FTZ by UPLC-ESI-MS analysis was obtained. Supplement Tables. Supplement Table 1: UPLC flow phase gradient elution procedure. Supplement Table 2: FTZ serum constituents. [file 6378786.f1.zip › 6378786.f1/Supplement Tables_ECAM_2600256.docx]

Supplement Tables

**Supplement Table 1 UPLC flow phase gradient elution procedure**

| time  (min) | Flow rate (mL/min) | A (%) | B (%) |
| --- | --- | --- | --- |
| 0.00 | 0.40 | 2.00 | 98.00 |
| 1.60 | 0.40 | 5.00 | 95.00 |
| 7.60 | 0.40 | 20.00 | 80.00 |
| 9.60 | 0.40 | 20.00 | 80.00 |
| 14.60 | 0.40 | 35.00 | 65.00 |
| 17.60 | 0.40 | 80.00 | 20.00 |
| 18.00 | 0.40 | 100.00 | 0.00 |
| 18.40 | 0.40 | 100.00 | 0.00 |
| 18.60 | 0.40 | 2.00 | 98.00 |
| 20.60 | 0.40 | 2.00 | 98.00 |

**Supplement Table 2 FTZ serum constituents**

| Components  Hydroxyl-palmatine  Thalifendine  Columbamine  Epiberberine  Coptisine  Jatrorrhizine  Berberrubine  Palmatine  Berberine  Dehydrocorydaline  13-Methylberberine  Jatrorrhizine3-O-β-D-glucuronide  Magnoflorine | Molecular weight  368  322  338  336  320  338  322  352  336  366  350  514  342 | origin  in serum and in preparation  in serum and in preparation  in serum and in preparation  in serum and in preparation  in serum and in preparation  in serum and in preparation  in serum and in preparation  in serum and in preparation  in serum and in preparation  in serum and in preparation  in serum and in preparation  in serum only  in serum and in preparation | |  |  |
| --- | --- | --- | --- | --- | --- |
| Notoginsenoside R_1_  Ginsenoside Rg_1_  Ginsenoside Rh_1_  Ginsenoside F_1_  Ginsenoside Rb_1_  Ginsenoside Rd  Protopanaxatriol  Ginsenoside Rg_3_  25-hydroxy-GinsenosideRh_1_  25-hydroxy-GinsenosideF_1_ | 932  800  638  638  1108  946  476  784  656  656 | | in serum and in preparation  in serum and in preparation  in serum and in preparation  in serum and in preparation  in serum and in preparation  in serum and in preparation  in serum only  in serum only  in serum only  in serum only | |  |
| Salvianolic acid B | 154 | | in serum and in preparation | | |
